# It Is Better to Be Semi-Regular When You Have a Low Degree

**DOI:** 10.3390/e26121014

**Published:** 2024-11-23

**Authors:** Theodore Kolokolnikov

**Affiliations:** Department of Mathematics and Statistics, Dalhousie University Halifax, Halifax, NS B3H 3J5, Canada; tkolokol@gmail.com

**Keywords:** algebraic connectivity, spectral gap, diffusion on graphs, computational graph theory, extremal graph theory

## Abstract

We study the algebraic connectivity for several classes of random semi-regular graphs. For large random semi-regular bipartite graphs, we explicitly compute both their algebraic connectivity as well as the full spectrum distribution. For an integer $d\in \left[3,7\right]$, we find families of random semi-regular graphs that have higher algebraic connectivity than random *d*-regular graphs with the same number of vertices and edges. On the other hand, we show that regular graphs beat semi-regular graphs when d≥8. More generally, we study random semi-regular graphs whose average degree is *d*, not necessarily an integer. This provides a natural generalization of a *d*-regular graph in the case of a non-integer d. We characterize their algebraic connectivity in terms of a root of a certain sixth-degree polynomial. Finally, we construct a small-world-type network of an average degree of 2.5 with relatively high algebraic connectivity. We also propose some related open problems and conjectures.

## 1. Introduction

Algebraic connectivity (AC; also called the spectral gap) of a graph is a fundamental property that measures how fast information diffuses throughout the graph [1,2]. It corresponds to the second smallest eigenvalue of the graph Laplacian matrix (the smallest eigenvalue is always zero). In many applications, it is desirable to maximize the algebraic connectivity (i.e., speed of diffusion) subject to certain constraints; this and related problems have a long history [3,4,5,6,7,8,9]. For example, in communications, the “cost” of a network increases with the number of links. It is therefore desirable to have as few edges as possible, while at the same time maximizing the algebraic connectivity. This leads to a natural question: *For a fixed average degree d*, *what is the graph that maximizes algebraic connectivity as the number of vertices* n→∞?

Numerous papers address various aspects of this question, see e.g., [6,7,8,9]. This is one of those situations where the best answer is elusive [10], but a decent answer can be found relatively quickly [3,4]. Graphs with high algebraic connectivity are related to expander graphs, and are important in many applications [11,12]. In this paper, we study the algebraic connectivity of *sparse* random graphs in the case when the number of edges *m* scales linearly with the number of vertices *n*, i.e., m=O(n). In other words, we fix the average degree d=2m/n=O(1) while letting n→∞.

It is well known that a random Erdos–Renyi graph (where *m* edges are taken at random) needs O(nlogn) edges to be fully connected [13,14], and as such, they are poor candidates for maximizing algebraic connectivity in this sparse regime (since the AC of a disconnected graph is zero). A good candidate is a *d*-regular graph for which every vertex has a degree *d*. It is well known that random regular *d*-graphs have an algebraic connectivity that is asymptotic to $\mu ∼d-2\sqrt{d-1}$ as n→∞ with fixed *d* [15,16,17,18,19]. For integer d≥3, this quantity is strictly positive, which assures that these graphs have good expander properties [20,21]. A natural question is whether one can perform better than a regular graph, but without “too much work” (in, say, O(n) time). In this paper, we give an affirmative answer when d≤7: we introduce a class of semi-regular random graphs (whose vertices have a degree of either $d_{1}$ or $d_{2}\neq d_{1}$), which is as easy to construct as random *d*-regular graphs, but which has better AC.

Another question is whether “expander-type” graphs (which we define to be graphs with AC bounded away from 0 as n→∞) are possible when the average degree *d* is less than 3, with n→∞. For 2<d<3, the answer is yes, and it is provided by semi-regular graphs of degrees 2 and 3, whose average degree is 2+p (with 0<p<1). We show that the AC of such graphs is asymptotic to $\mu ∼p^{2}/4$ in the limit of small *p* and large *n*, with n→∞ independent of *p* (the answer is no when d≤2, since any graph needs at least n−1 edges to be connected).

Our first result is on random semi-regular *bipartite* graphs, where each vertex has a degree of either $d_{1}$ or $d_{2}$, and we compute their asymptotic AC in the limit n→∞. In particular, we will exhibit a family of semi-random bipartite graphs having the same average degree as random *d*-regular graphs, but which have a higher algebraic connectivity when d≤7.

Before stating our result, let us define what we mean by such graphs. Consider a bipartite graph with one part having $n_{1}$ vertices of degree $d_{1}$, and the second part having $n_{2}$ vertices of degree $d_{2}$, such that every edge is between these two parts, and no edges are within each part. Then, $n_{1}d_{1}=n_{2}d_{2}$. Such a graph has $n=n_{1}+n_{2}$ vertices so that

(1) $$n_{1}=\frac{d_{2}}{d_{1}+d_{2}}n,\;\;\;n_{2}=\frac{d_{1}}{d_{1}+d_{2}}n,$$

and therefore, the average degree is

(2) $$d=\frac{2d_{1}d_{2}}{d_{1}+d_{2}}.$$

We call such a graph a $\left(d_{1},d_{2}\right)$ semi-regular bipartite graph. We now introduce the following random model.

**Figure 1:**
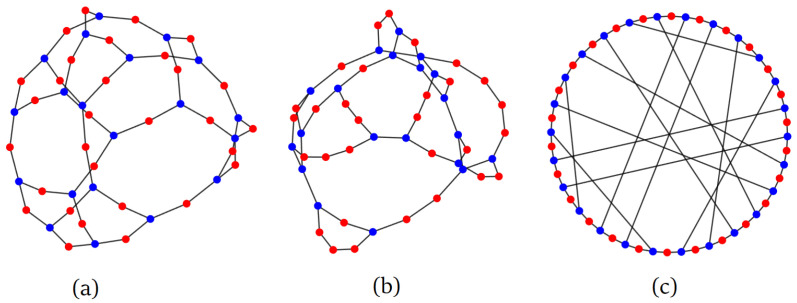
Types of graphs considered in this work. Colour is used for emphasis, with blue vertices having degree 3 and red vertices having degree 2. (**a**) Random semi-regular bipartite graph with $\left(d_{1},d_{2}\right)=\left(2,3\right)$. (**b**) Random semi-regular graph with $\left(p,d_{1},d_{2}\right)=\left(0.4,2,3\right).$ Both graphs have the same average degree of d=2.4. (**c**) Small-world network consisting of a ring and with edges added at random between the odd-numbered vertices. It has an average degree of d=2.5.

**Random semi-regular bipartite (RSRB) graph model**: Take two bags. In the first bag, put $d_{1}$ copies of $n_{1}$ vertices labeled $1\ldots n_{1}.$ In the second bag, put $d_{2}$ copies of $n_{2}$ vertices labeled $n_{1}+1\ldots n_{1}+n_{2}.$ Start with an empty graph of $n_{1}+n_{2}$ vertices. Then, randomly pick two vertices without replacement—one from each bag—and add an edge between them to the graph. Repeat until the bags are empty. Refer to Figure 1a and the Matlab code in Appendix A.

We remark that this model (and the theory below) generally allows for multiple edges. These can be eliminated through a random rewiring postprocessing step. (Given a multiple edge (a,b) or a self-loop (a=b), choose another edge $\left(c,d\right)$ at random. Then, replace edges $\left(a,b\right)$ and $\left(c,d\right)$ by edges $\left(a,c\right)$ and $\left(b,d\right)$. Repeat until all multiple edges/loops are eliminated. This operation preserves the degree distribution and edge count). Multi-edges/loops appear to be sufficiently rare that the postprocessing step does not affect the asymptotic results in the large *n* limit, as confirmed via direct numerical simulations. We now state our main results for RSRB graphs.

**Main Result** **1.** *Consider a $\left(d_{1},d_{2}\right)$ RSRB graph. In the limit n→∞, its spectrum density is asymptotic to*

(3) $$\rho \left(x\right)=\left\{\begin{array}{c} \frac{1}{\pi }\frac{d_{1}d_{2}}{d_{1}+d_{2}}\frac{\sqrt{\left(x^{2}-r_{-}^{2}\right)\left(r_{+}^{2}-x^{2}\right)}}{\left(d_{1}d_{2}-x^{2}\right)\left|x\right|},\;\;\left|x\right|\in \left(r_{-},r_{+}\right)\\ \frac{\left|d_{2}-d_{1}\right|}{d_{1}+d_{2}}\delta \left(x\right),\;\;\left|x\right|<r_{-}\\ 0,\;\;\;\left|x\right|>r_{+} \end{array}\right.$$

*where δ is the Dirac-delta function and*

(4) $$r_{\pm }={\left(d_{1}+d_{2}-2\pm \sqrt{{\left(d_{1}+d_{2}-2\right)}^{2}-{\left(d_{2}-d_{1}\right)}^{2}}\right)}^{1/2}.$$

*In other words, the number of eigenvalues inside any interval $\left(a,b\right)$ is asymptotic to $\int_{a}^{b}\rho \left(x\right)dx$ as n→∞.*

*Moreover, its algebraic connectivity is asymptotic to*

(5) $$\mu ∼\frac{d_{1}+d_{2}}{2}-{\left({\left(\frac{d_{2}-d_{1}}{2}\right)}^{2}+r_{+}^{2}\right)}^{1/2},\;\;\;n\gg 1.$$

**Figure 2:**
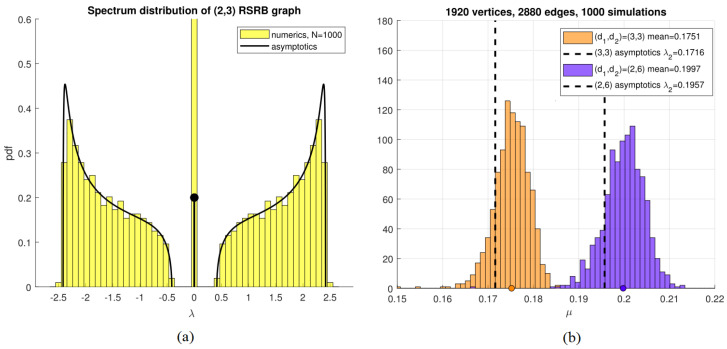
(**a**) Full spectrum of random semi-regular bipartite graph with $\left(d_{1},d_{2}\right)=\left(2,3\right)$. Numerics correspond to the histogram of eigenvalues of one of such a graph with 1000 vertices, computed numerically using Matlab. Asymptotics correspond to Formula (3). The height of the lollipop corresponds to the weight delta function at the origin. (**b**) Comparison of algebraic connectivity between $\left(3,3\right)$ regular bipartite, $\left(2,6\right)$ semi-regular bipartite graphs, and the asymptotic theory. The two classes have the same number of vertices and edges, and $\left(2,6\right)$ is 15% better than (3,3) (both for asymptotics and numerics).

Figure 2a shows the shape of the distribution (3) for the case $d_{1}=2,\;\;d_{2}=3$ (having an average degree of d=2.4), and compares it to an numerical histogram of eigenvalues of a 1000-vertex graph for this case. Very good agreement is observed.

Note that in the case $d_{1}=d_{2}$, Formula (3) reduces to the well-known Mckay distribution of the spectrum of a regular graph [15]:

(6) $$\rho \left(x\right)=\left\{\begin{array}{c} \frac{1}{\pi }\frac{d}{2}\frac{\sqrt{4\left(d-1\right)-x^{2}}}{d^{2}-x^{2}},\;\;\left|x\right|<2\sqrt{d-1}\\ 0,\;\;\left|x\right|>2\sqrt{d-1} \end{array}\right..$$

Moreover, Formula (5) simplifies to the classical result $\mu ∼d-2\sqrt{d-1}$ for the algebraic connectivity of a random regular graph of *d* vertices (this also shows that a random regular graph and random bipartite regular graphs have the same algebraic connectivity, at least to leading order in *n*).

Consider a random cubic graph $\left(d_{1}=d_{2}=3\right)$ and contrast it with an RSRB graph with $d_{1}=2,\;d_{2}=6$. Both cases have the same average degree of d=3. Formula (5) gives the asymptotic values of μ∼0.17157 for the former, and μ∼0.19577, for the latter. *Thus, the*$\left(2,6\right)$ *RSRB graph is about 15% better than a random cubic graph with respect to its expander properties, while having the same number of vertices and edges.* Figure 2b shows a histogram for μ of 1000 randomly constructed such graphs, comparing these two cases with n=2000. Very good agreement between numerics and asymptotics is observed in both cases.

More generally, the following table shows all possible combinations of $d_{1},d_{2}$ such that $d=\frac{2d_{1}d_{2}}{d_{1}+d_{2}}$ is an integer between 3 and 8, and the corresponding value of μ (5).
All RSRB graphs with integer average degree d=3,…,8*d*345678$d_{1}$3243536474865$d_{2}$364651561272881220$\mu_{\mathrm{asympt}}$0.1715**0.1957**0.5358**0.5535**1**1.0890**1.5278**1.5587**2.1010**2.1435****2.7084**2.68872.6671$\mu_{\mathrm{numerics}}$0.1780.2050.5530.5721.0271.1221.5651.5962.1502.2052.7662.7452.729std0.0060.0060.0110.0100.0150.0170.0180.0180.0210.0200.0260.0220.022diff %3.8%4.7%3.1%3.2%2.7%3.0%2.4%2.4%2.3%2.87%2.1%2.1%2.2%

The row $\mu_{\mathrm{asympt}}$ is the asymptotic formula given by (5). Row $\mu_{\mathrm{numerics}}$ corresponds to Monte Carlo simulations of μ. It shows the average μ for 200 randomly chosen RSRB graphs with n=1000 edges. The row “diff %” is $\frac{\mu_{\mathrm{numerics}}-\mu_{\mathrm{asympt}}}{\mu_{\mathrm{asympt}}}\times 100$. Uniformly good agreement between asymptotics and numerics is observed.

Parameters with higher AC are shown in bold. For d≤7, the RSRB graph with $d_{1}\neq d_{2}$ has a higher algebraic connectivity than the *d*-regular graph. On the other hand, for d≥8, *d*-regular graphs excel.

It is interesting to note that for any integer d≥3, Equation (2) always has a solution with integers $2\leq d_{1}<d_{2}.$ When *d* is prime, this solution is unique and is given by $d_{1}=\left(d+1\right)/2$, $d_{2}=d\left(d+1\right)/2.$ More generally, the number of such solutions is precisely the number of Pythagorean triples of leg *d* (sequence A046079 in OEIS).

The RSRB graphs above have a constraint $n_{1}d_{1}=n_{2}d_{2}.$ In particular, the minimum attainable average degree of such graphs is d=2.4, corresponding to $\left(d_{1},d_{2}\right)=\left(2,3\right).$ We can remove this constraint by instead introducing the probability of having degree $d_{1}$ or $d_{2}$ as follows.

**Random semi-regular (RSR) model:** Given $p,d_{1},d_{2}$, and *n*, let $n_{1}=\left\lfloor \left(1-p\right)n\right\rfloor$ and let $n_{2}=n-n_{1}$. In the same bag, put $d_{1}$ copies of $n_{1}$ vertices labeled $1\ldots n_{1}$ and $d_{2}$ copies of $n_{2}$ vertices labeled $n_{1}+1\ldots n.$ Create edges by drawing two vertices from the bag at random (without replacement), until only one or zero vertices are left in the bag. See Appendix A for the Matlab code.

An example of an RSR graph is shown in Figure 1b. Note that such a graph has an average degree of $d=\left(1-p\right)d_{1}+pd_{2}$. We have the following.

**Main Result** **2.** *Consider a $\left(p,\;d_{1},d_{2}\right)$ random semi-regular graph. Let*

(7) $$F\left(R,x\right)=x\left(d_{2}-d_{1}\right)\left(1-Rx\right)p+\left(Rx^{2}\left(d_{2}-1\right)-1\right)\left(R^{2}x^{2}\left(d_{1}-1\right)+Rx\left(d_{2}-d_{1}\right)-R+1\right).$$

*Let x be the smallest root of the system F=0=∂F/∂R. Then, in the limit n→∞, the AC is given by $\mu =d_{2}-1/x.$*

In general, eliminating *R* from the system F=0=∂F/∂R is a straightforward computer algebra computation using a resultant, and yields in a sixth-degree polynomial for *x*. It is too complex to present here for the general $d_{1},d_{2}$—see Appendix A for the Maple code. In the case $d_{1}=2,d_{2}=3$, the RSR graph has an average degree 2+p, and μ is the smallest root of

(8) $$0=\mu \left(\mu -4\right)\left(\mu^{2}-4\mu -1\right)+2\mu \left(3\mu^{3}-33\mu^{2}+89\mu -19\right)p+\left(-15\mu^{2}-30\mu +1\right)p^{2}+8p^{3}.$$

**Figure 3:**
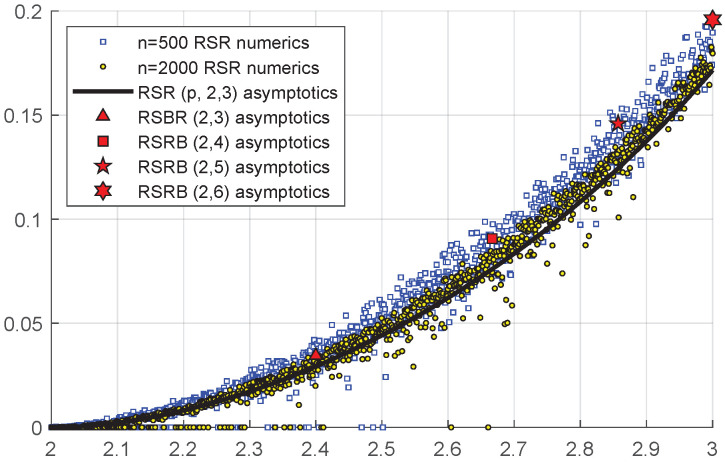
Comparison between RSR, RSRB asymptotics, and RSR numerics, with average degree 2<d≤3. Numerics represent the AC of 1000 randomly chosen RSR graphs with p∈(0,1) and $\left(d_{1},d_{2}\right)=\left(2,3\right)$. Here, μ is plotted against d=2+p. Asymptotics for RSR correspond to roots of (8). Asymptotics for RSRB are given by (5).

Figure 3 compares the μ given by (8) with numerical computations of μ for randomly chosen $\left(p,2,3\right)$ RSR graphs. Note that the numerical result approaches the asymptotic value of μ as the number of edges *n* is increased.

The following table gives the value of μ for several choices of $p,d_{1},d_{2}$, for which the average degree d=4, and a comparison with numerics is shown.
RSRmodelwithd=4$d_{1}$433222$d_{2}$456567*p*
0.51/32/30.50.4$\mu_{\mathrm{asympt}}$**0.5359**0.442610.391620.33330.253520.20748$\mu_{\mathrm{numerics}}$0.5510.4880.4510.2860.2170.174std0.0100.0200.0220.0620.0510.045diff %2.8%10%15%−14%−14%−19%

Here, $\mu_{\mathrm{asympt}}$ is as given in the Main Result 2; $\mu_{\mathrm{numerics}}$ is the average of 200 simulations with n=1000. As apparent from this table, the RSR model performs worse than the RSRB of average degree 4 (including 4-regular graphs). This appears to be true for any average degree *d*. However, one advantage of RSR graphs is that they produce graphs with an average degree 2<d<3, with μ bounded away from zero as n→∞. The smallest average degree that the RSRB model can have is 2.4, corresponding to $\left(d_{1},d_{2}\right)=\left(2,3\right).$ In fact, (8) shows that $\mu ∼p^{2}/4$ when the average degree is 2+p with $d_{1}=2,d_{2}=3$, and 0<p≪1.

## 2. Derivation of Main Results

**Derivation of spectral density (3).** Following [15,22], we use the trace method. It is based on the fact that $\mathrm{trace}\left(A^{s}\right)=\sum_{j=1}^{n}\lambda_{j}^{s}$. Define $\phi_{s}=\frac{1}{n}\mathrm{trace}\left(A^{s}\right).$ Then, $\phi_{s}$ represents the average number of closed walks of length *s* on the graph whose adjacency matrix is *A*. In the limit n→∞, the eigenvalue distribution therefore satisfies

(9) $$\int x^{s}\rho \left(x\right)dx=\phi_{s}.$$

As in [15,22], the derivation consists of (a) computing $\phi_{s};$ and (b) inverting the integral Equation (9) to determine ρ(x).

To compute $\phi_{s}$, we write

$$\phi_{s}=\frac{d_{1}}{d_{1}+d_{2}}\phi_{A,s}+\frac{d_{2}}{d_{1}+d_{2}}\phi_{B,s},$$

where $\phi_{A,s}$ is the number of closed walks of length *s* starting from a vertex of degree $d_{2}$, and $\phi_{B,s}$ is the number for the vertices of degree $d_{1}.$ A key insight [15] that allows for the asymptotic determination of $\phi_{A,s}$ and $\phi_{B,s}$ is that, locally, a large random regular graph looks like a tree because the probability of encountering a cycle of length *s* is incredibly small as n→∞ (for fixed *s*). The same is true of semi-regular random graphs. For the RSRB graphs, each successive level alternates between vertices of degree $d_{1}$ and $d_{2}.$ We therefore decompose these trees as illustrated in the following diagram, for the cases $d_{1}=2$ and $d_{2}=3$.

$$\begin{array}{c} d_{1}=2,\;\;d_{2}=3 \end{array}$$

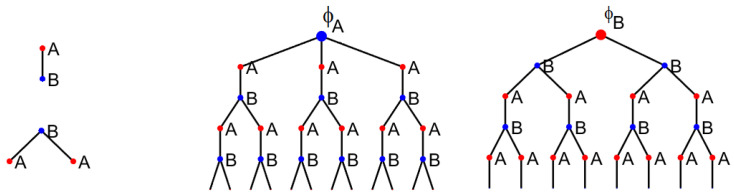


Here, *A* represent the child nodes of degree $d_{1}$ and *B* child nodes of degree $d_{2}$. Accordingly, $\phi_{A,s}$ and $\phi_{B,s}$ satisfy the recursion

(10) $$\begin{array}{cc} \phi_{A,s} & =d_{2}\sum_{j=0}^{s-2}\phi_{A,j}A_{s-2-j},\;\;\;\;\phi_{B,s}=d_{1}\sum_{j=0}^{s-2}\phi_{B,j}B_{s-2-j},\\ A_{s} & =\left(d_{1}-1\right)\sum_{j=0}^{s-2}A_{j}B_{s-2-j},\;\;\;\;\;B_{s}=\left(d_{2}-1\right)\sum_{j=0}^{s-2}B_{j}A_{s-2-j}, \end{array}$$

with $A_{0}=B_{0}=\phi_{A,0}=\phi_{B,0}=1$. Let $A\left(x\right)=\sum A_{s}x^{s}$ be the generating function for $A_{s}$ and similarly for $B,\;\phi_{A},\;\phi_{B},\;\phi .$ From recursion relations (10), the corresponding generating functions satisfy   

(11) $$\begin{array}{cc} A & =1+\left(d_{1}-1\right)x^{2}AB,\\ B & =1+\left(d_{2}-1\right)x^{2}BA,\\ \phi_{A} & =1+d_{2}x^{2}\phi_{A}A,\\ \phi_{B} & =1+d_{1}x^{2}\phi_{B}B,\\ \phi & =\frac{d_{1}}{d_{1}+d_{2}}\phi_{A}+\frac{d_{2}}{d_{1}+d_{2}}\phi_{B}. \end{array}$$

Solving (11), while keeping in mind that $\phi \left(0\right)=1$, we find that

$$\phi \left(x\right)=\frac{d_{1}d_{2}}{d_{1}+d_{2}}\frac{\frac{d_{1}+d_{2}}{d_{1}d_{2}}-\sqrt{{\left(d_{2}-d_{1}\right)}^{2}x^{4}+\left(4-2d_{1}-2d_{2}\right)x^{2}+1}-1}{d_{1}d_{2}x^{2}-1}.$$

In principle, $\phi_{s}$ can be computed from ϕ(x) by Taylor-expanding near the origin; the first few terms are $\phi_{0}=1,\;\phi_{2}=2\frac{d_{1}d_{2}}{d_{1}+d_{2}},\;\phi_{4}=2\frac{d_{1}d_{2}}{d_{1}+d_{2}}\left(d_{1}+d_{2}-1\right)$. In [15], an explicit computation of $\phi_{s}$ was combined with Tchebychev polynomials to compute the McKay distribution for *d*-regular random graphs. Here, we use a simpler and more powerful technique using complex variables introduced in [23].

Write $\phi_{s}$ using Cauchy’s integral formula as

$$\phi_{s}=\int z^{-s-1}\phi \left(z\right)\frac{dz}{2\pi i},$$

where the integration is around a circle $\left|z\right|=\varepsilon$, with a sufficiently small ε to avoid any branch cuts of ϕ. Using the fact that ϕ(z) is even, we then have

$$\begin{array}{cc} \phi_{s} & =\frac{1}{\pi }\int_{0}^{\pi }\varepsilon^{-s}\phi \left(\varepsilon e^{i\theta }\right)e^{-si\theta }d\theta \\ \; & =\frac{i}{\pi }\int_{-1/\varepsilon }^{1/\varepsilon }\frac{1}{x}\phi \left(\frac{1}{x}\right)x^{s}dx. \end{array}$$

Taking ε→0 and recalling (9), we obtain

$$\int \rho \left(x\right)x^{s}dx=\frac{i}{\pi }\int_{-\infty }^{\infty }\frac{1}{x}\phi \left(\frac{1}{x}\right)x^{s}dx.$$

This yields the formula for ρ, namely

(12) ρ(x)=−1πIm1xϕ1x.

This formula was derived in [23] using an equivalent technique (Stieltjes inversion). Next, we compute

(13) $$\frac{1}{x}\phi \left(\frac{1}{x}\right)=\frac{1}{x}\frac{d_{1}d_{2}}{d_{1}+d_{2}}\frac{\frac{d_{1}+d_{2}}{d_{1}d_{2}}x^{2}-\sqrt{\left(x^{2}-r_{-}^{2}\right)\left(x^{2}-r_{+}^{2}\right)}-x^{2}}{d_{1}d_{2}-x^{2}},$$

where $r_{\pm }$ is given by (4). It follows that Im1xϕ1x=0 for $\left|x\right|\notin \left(r_{-},r_{+}\right)$ and x≠0. On the other hand, when $\left|x\right|\in \left(r_{-},r_{+}\right),$ we obtain

(14) $$\rho \left(x\right)=\frac{1}{\pi }\frac{1}{\left|x\right|}\frac{d_{1}d_{2}}{d_{1}+d_{2}}\frac{\sqrt{\left(r_{+}^{2}-x^{2}\right)\left(x^{2}-r_{-}^{2}\right)}}{d_{1}d_{2}-x^{2}},\;\;\left|x\right|\in \left(r_{-},r_{+}\right).$$

This recovers Formula (3) except for the delta mass at the center, which is due to the singularity there. To compute the weight of the delta mass, one can integrate the overall density and impose that ∫ρ=1. While the exact integration of (14) is possible, it is easier and more instructive to compute the number of zero eigenvalues directly, as follows.

Suppose that $d_{2}>d_{1}.$ Label the components of the eigenvector $x_{1}\ldots x_{n_{1}}$ and $y_{1}\ldots y_{n_{2}}$, corresponding to vertices of degree $d_{1}$ and $d_{2}$, respectively. A zero eigenvalue satisfies $\sum_{x_{j}\in nbr\left(y_{k}\right)}x_{j}=0$, $k=1\ldots n_{2}$ and $\sum_{y_{j}\in nbr\left(x_{k}\right)}y_{j}=0$, $k=1\ldots n_{1}.$ Look for solutions of the form where $y_{j}=0$, $j=1\ldots n_{2}.$ This corresponds to solving $n_{2}$ linear equations $\sum_{x_{j}\in nbr\left(y_{k}\right)}x_{j}=0$, for the $n_{1}$ unknown $x_{1}\ldots x_{n_{1}}.$ This is an under-determined system, since $n_{2}<n_{1}.$ Generically, it has $n_{1}-n_{2}$ independent solutions. Therefore, the weight of the delta function at zero should be $\frac{n_{1}-n_{2}}{n}.$ Using (2) yields $\frac{n_{1}-n_{2}}{n}=\frac{d_{2}-d_{1}}{d_{1}+d_{2}}$, which recovers the weight of the delta function in (3). We also verified using computer algebra that this indeed agrees with the full integration of (14), namely, that $\int_{r_{-}}^{r_{+}}\rho \left(x\right)dx=\frac{d_{1}}{d_{1}+d_{2}}$, $d_{2}>d_{1}$, so that, indeed, $\int_{-\infty }^{\infty }\rho =1$. This completes the derivation of the spectral density (3).

Finally, another derivation of the delta weight, as pointed out by James Mingo, is to use Proposition 3.8 in [24]. It says that if $\frac{1}{x}\phi \left(\frac{1}{x}\right)$ has a singularity at x=a, then the associated measure ρ(x) has a delta mass at x=a, whose weight is given by $\lim_{x\to a}\left|\left(x-a\right)\frac{1}{x}\phi \left(\frac{1}{x}\right)\right|.$ Here, a=0 and the mass weight is then given by $\lim_{x\to 0}\left|\phi \left(\frac{1}{x}\right)\right|=\frac{d_{1}d_{2}}{d_{1}+d_{2}}\frac{\sqrt{r_{-}^{2}r_{+}^{2}}}{d_{1}d_{2}}=\frac{\left|d_{2}-d_{1}\right|}{d_{1}+d_{2}}$ in agreement with direct computation. ▪

**Derivation of AC formula for RSRB** (5). Next, we derive the formula for algebraic connectivity (5). For *d*-regular graphs, the Laplacian graph is given by dI−A, where *I* is the identity and *A* is the adjacency matrix. This allows us to characterize the AC in terms of the second-largest eigenvalue λ of *A*: μ=d−λ. Of course, this is not true if the graph is not regular. The trick is to **regularize the graph by adding enough self-loops to vertices until all vertices have the same degree**. Each self-loop counts as a single additional edge (i.e., adding a loop to vertex *j* corresponds to adding “1” to the *j*-th diagonal entry of the associated adjacency matrix) and crucially, adding self-loops does not change the Laplacian of the graph.

For a $\left(d_{1},d_{2}\right)$ semi-regular graph, assume that $d_{1}<d_{2}$ and add $d_{2}-d_{1}$ loops to all vertices of degree $d_{1}$. This results in a $d_{2}$-regular graph with loops. As before, let $\phi_{s}$ be the number of closed walks of size *s*. Let λ be the *local expansion rate*, that is, the rate at which $\phi_{s}$ grows: $\lambda =\lim_{s\to \infty }\phi_{s+1}/\phi_{s}$. Then, by analogy to regular graphs, the AC is given by $\mu =d_{2}-\lambda$. To compute λ, we decompose closed loops similarly to (11). The decomposition now has loops as illustrated below.

$$\begin{array}{c} d_{1}=2,\;d_{2}=3 \end{array}$$

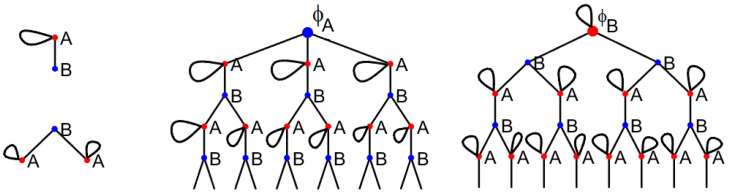


Correspondingly, the associated generating functions satisfy

(15) $$\begin{array}{cc} A & =1+\left(d_{2}-d_{1}\right)xA+\left(d_{1}-1\right)x^{2}AB,\;\;\;\\ B & =1+\left(d_{2}-1\right)x^{2}BA,\\ \phi_{A} & =1+d_{2}x^{2}\phi_{A}A,\\ \phi_{B} & =1+x\left(d_{2}-d_{1}\right)\phi_{B}+d_{1}x^{2}\phi_{B}B,\\ \phi & =\frac{d_{1}}{d_{1}+d_{2}}\phi_{A}+\frac{d_{2}}{d_{1}+d_{2}}\phi_{B}. \end{array}$$

To determine AC, it suffices to determine the growth rate of $\phi_{s}$. This growth rate is in turn determined by the locations of singularities in the associated generating functions [25]. In particular, if $\sum \phi_{s}z^{s}=g\left(z\right)+h\left(z\right){\left(z-r\right)}^{p}$, where *p* is non-integer and g,h analytic, then $\phi_{s}$ grows with the rate $r^{-1},$ in other words, $\phi_{s+1}/\phi_{s}\to r^{-1}$ as s→∞. The location of the singularity can be found without solving the full system, and corresponds to the smallest root of the discriminant of the system. To illustrate this, consider the Catalan numbers $c_{n}=\frac{1}{n+1}\left(\begin{array}{c} 2n\\ n \end{array}\right)$, whose generating function satisfies $c=1+xc^{2}.$ Explicitly, $c\left(x\right)=\frac{1-\sqrt{1-4x}}{2x}$, and has a singularity at x=1/4, corresponding to the zero of the discriminant of the quadratic $xc^{2}-c+1=0.$ It follows that $c_{n+1}/c_{n}\to 4$ as n→∞ (indeed, more precise asymptotics $c_{n}∼\frac{1}{\sqrt{\pi }}4^{n}n^{-3/2}$ can also be derived from its generating function, although here, we only need the growth rate).

Let us now return to system (15). Eliminating *B*, we find that *A* satisfies a quadratic:

$$\left(d_{2}-1\right)\left(x\left(d_{2}-d_{1}\right)-1\right)x^{2}A^{2}+\left[x^{2}\left(d_{2}-d_{1}\right)-x\left(d_{2}-d_{1}\right)+1\right]A-1=0.$$

Setting its discriminant to zero, we obtain a quartic polynomial for *x*, namely

(16) $${\left(d_{1}-d_{2}\right)}^{2}x^{4}+2\left(d_{1}+d_{2}-2\right)\left(d_{2}-d_{1}\right)x^{3}+\left({\left(d_{1}-d_{2}\right)}^{2}-2\left(d_{1}+d_{2}-2\right)\right)x^{2}+2\left(d_{1}-d_{2}\right)x-1=0.$$

The expansion rate is then λ=1/x, so that $\mu =d_{2}-1/x$. Substituting $x=1/(\mu -d_{2})$ into (16), one obtains a fourth-degree polynomial for μ:

$$0=\mu^{4}-2\left(d_{1}+d_{2}\right)\mu^{3}+\left({\left(d_{1}+d_{2}\right)}^{2}+2d_{1}d_{2}-2d_{1}-2d_{2}+4\right)\mu^{2}+2\left(d_{1}+d_{2}\right)\left(d_{1}+d_{2}-2-d_{1}d_{2}\right)\mu +{\left(d_{1}d_{2}-d_{1}-d_{2}\right)}^{2}.$$

Shift this polynomial to eliminate the $\mu^{3}$ term by substituting $\mu =y+\frac{d_{1}+d_{2}}{2}.$ Almost like magic, it simultaneously eliminates the $\mu^{1}$ term, resulting in a quadratic for $y^{2}$:

$$0=y^{4}+\left(4-2\left(d_{1}+d_{2}\right)-{\left(d_{2}-d_{1}\right)}^{2}/2\right)y^{2}+\frac{{\left(d_{2}-d_{1}\right)}^{2}\left(d_{1}+d_{2}\right)}{2}+\frac{{\left(d_{2}-d_{1}\right)}^{4}}{16}.$$

Solving for *y* using the quadratic formula yields the solution (5). ▪

**Derivation of Result 2.** Again, we compute the average number of closed walks of length *s*, $\phi_{s}.$ As before, each vertex looks like a tree locally. Each child in this tree has the probability $\left(1-p\right)$ of having degree $d_{1}$, and probability *p* of having degree $d_{2}.$ As in the derivation of (5), assume that $d_{1}\leq d_{2}$, and add $d_{2}-d_{1}$ self-loops to each vertex of degree $d_{1}.$ The walks on these trees then decompose according to the following diagram (here given in the case $d_{1}=2,\;d_{2}=3$).

$$\begin{array}{c} d_{1}=2,\;\;d_{2}=3 \end{array}$$

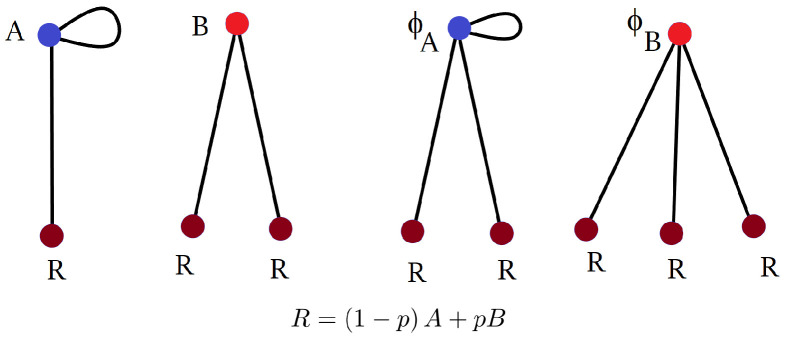


The corresponding generating function $\phi \left(x\right)=\sum \phi_{s}x^{s}$ solves the equations:

(17) $$\begin{array}{cc} A & =1+x\left(d_{2}-d_{1}\right)A+\left(d_{1}-1\right)x^{2}AR\\ B & =1+\left(d_{2}-1\right)BR\\ R & =\left(1-p\right)A+pB\\ \phi_{A} & =1+x\left(d_{2}-d_{1}\right)\phi_{A}+d_{1}x^{2}\phi_{A}R\\ \phi_{B} & =1+d_{2}x^{2}\phi_{B}R\\ \phi & =\left(1-p\right)\phi_{A}+p\phi_{B}. \end{array}$$

Eliminating *A* and *B* yields a cubic F(R;x)=0 given by (7). The AC is then given by $\mu =d_{2}-1/x$, where *x* is the singularity of R(x) that is closest to the origin. By the implicit function theorem, this happens when $F_{R}=0.$ In other words, *x* satisfies F=0=∂F/∂R. ▪

## 3. Discussion and Open Problems

We computed the asymptotics of the AC for two models of semi-regular random graphs: either RSR or RSRB models. While the RSR model is shown to have a smaller AC than a random *d*-regular graph with the same average degree, we found that the RSRB model has a *higher* AC than *d*-regular when d≤7.

Ramunajan expander graphs are defined as being *d*-regular graphs [11,12] whose algebraic connectivity is at least $d-2\sqrt{d-1}$. As illustrated in Figure 2b (see also Figure 9 in [11] and the related table there), a random *d*-regular graph has a decent chance of being a Ramunajan graph (around 66% when d=3 as n→∞ according to simulations in [11]). We can extend the definition of Ramunajan expander graphs as being any graph of *average* degree *d* whose AC is at least $d-2\sqrt{d-1}.$ When d=3, an RSRB $\left(2,6\right)$ graph is actually a Ramunajan graph with very high probability as n→∞. This is illustrated in Figure 2b, where n≈2000. Out of 1000 of such randomly chosen graphs, only *one* had an AC less than $d-2\sqrt{d-1}=0.1716$.

**Figure 4:**
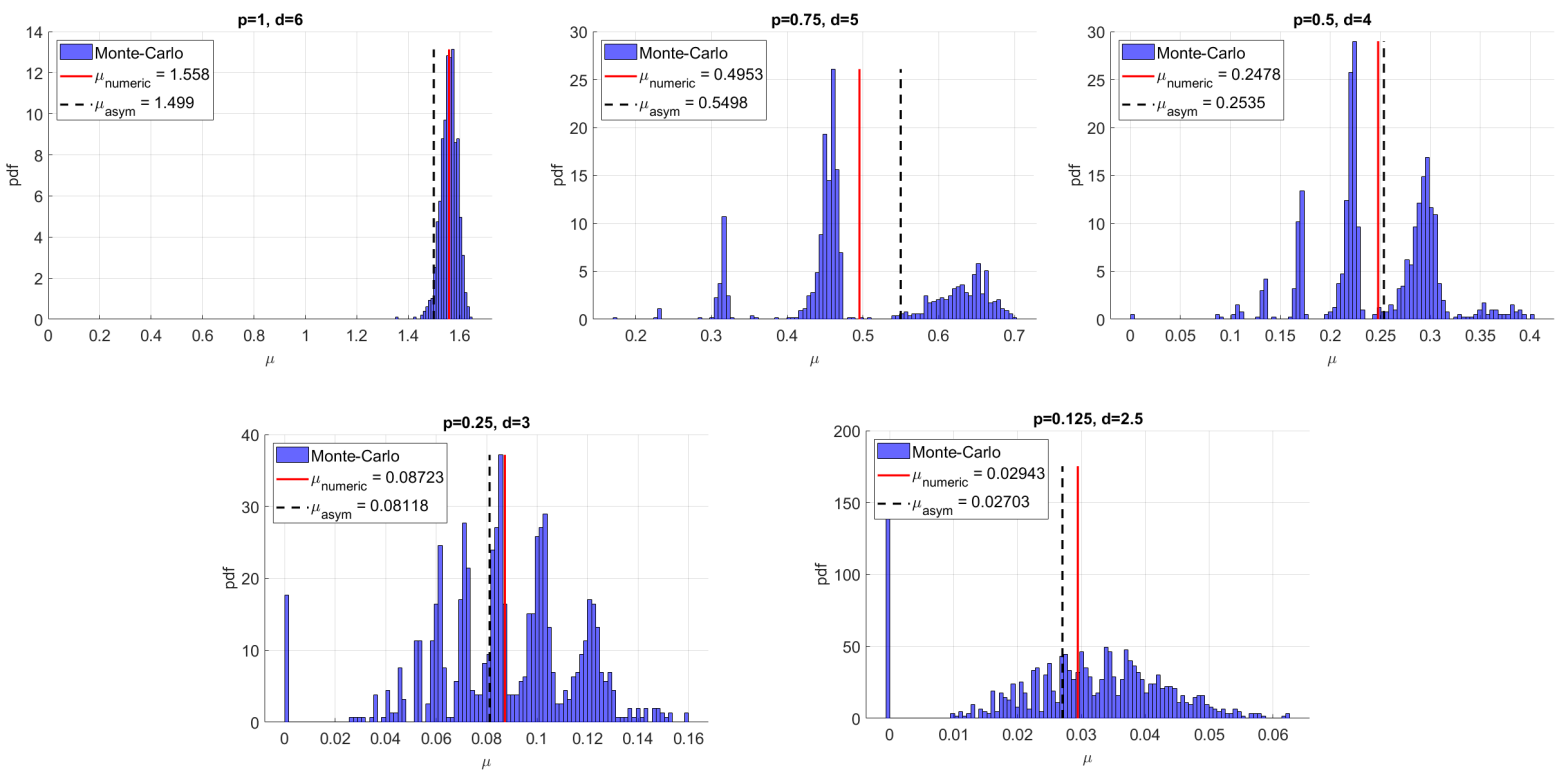
Distribution of AC for RSR graphs with $\left(d_{1},d_{2}\right)=\left(2,6\right)$ and with *p* as indicated. Average degree d=2+4p is also indicated. We used n=500 nodes and 1000 simulations. $\mu_{\mathrm{numeric}}$ is the average of the distribution, whereas $\mu_{\mathrm{asym}}$ is the asymptotics according to Main Result 2. Note the multi-peaked shape of the distribution, and the fact that the distribution does not concentrate around the mean when d≤5.

We relied on careful but formal power series computations. While the results were validated using numerics, a rigorous analysis of the results in this paper is an open problem. Related to this, it is an open question to estimate the accuracy of asymptotics as a function of n. In particular, the accuracy appears to be significantly degraded for the RSR graphs when $d_{1}$ and $d_{2}$ are far from each other. Take, for example, the case $\left(d_{1},d_{2}\right)=\left(2,6\right)$. Intriguingly, the distribution for AC appears to have multiple peaks and does not necessarily concentrate around the mean, as illustrated in Figure 4 (note that this does not appear to be the case for RSRB graphs as Figure 2b illustrates). We pose this as an open problem.

**Challenge** **1.** *Describe the full distribution of the AC, particularly for RSR graphs. Explain why it can be multi-peaked when $d_{1}\neq d_{2}$.*

Another class of graphs having high algebraic connectivity are the complete bipartite graphs $K_{b,n-b}$, which have AC μ=b (with b<n/2). The average degree of such a graph is asymptotic to d=2b as n→∞, so that μ∼d/2. This is in contrast to *d*-regular random graphs ($\mu ∼d-2\sqrt{d-1}$). As was noted in [7], $K_{d/2,n-d/2}$ have higher AC than the *d*-regular graphs (for the same asymptotical number of edges) provided that d<15 (since, in which case, this is the $d/2>d-2\sqrt{d-1}$ case) while the converse is true when d≥15. While complete bipartite graphs have a relatively high AC when d<15, they are also fragile, in the sense that removing any single edge causes the AC to decrease by one. Random graphs are more robust with respect to edge deletions as they seem to have some redundancy built-in.

Consider the limit of large $d_{2}$ for the RSRB model. From (2) and (5), one obtains that $d∼2d_{1}$ and $\mu ∼\frac{d}{2}-1$ as $d_{2}\to \infty .$ This is one less than the AC of the complete bipartite graph $K_{d/2,n-d/2}$ (which also has the average degree *d* as n→∞). More generally, let $\mu_{d_{1},d_{2}}$ be as in (5) and let $\mu_{d}=d-2\sqrt{d-1}$, where $d=2d_{1}d_{2}/\left(d_{1}+d_{2}\right)$ is the average degree. It can be shown that $\mu_{d_{1},d_{2}}<\mu_{d}$ for any $d_{1},d_{2}$ when d≥10. This discussion suggests the following question.

**Challenge** **2.** *Find a family of random graphs which has a higher algebraic connectivity than d-regular random graphs when the average degree d≥10. Explore if a more complex degree distribution (e.g., tri-regular) can be better than semi-regular for, say, d=3.*

There are several notions of graph connectivity, with AC being just one. Another notion is using the average of reliability polynomial [26,27] to compute the effect of edge deletions on graph connectivity. While the full reliability polynomial requires exponential time to compute, we performed the following simple experiment to quickly measure graph robustness. Start with a random 6-regular graph on 500 vertices (i.e., containing 1500 edges). Then, delete edges at random one by one until the graph becomes disconnected. Over 100 simulations, it took on average 460 edge deletions until the graph became disconnected (and in every instance, disconnectivity first occurred when a single vertex lost all of its edges). We then repeated this experiment with $\left(d_{1},d_{2}\right)=\left(4,12\right)$ RSRB graph with 500 vertices (which also contains 1500 edges, but has higher AC than a 6-regular random graph). It took on average 325 edge deletions until the disconnection was achieved. These preliminary experiments indicate that *d*-regular graphs are more reliable with respect to edge deletions than semi-regular bipartite graphs, even though the latter might have a higher AC. We state this as a conjecture.

**Conjecture** **1.** *For a given average degree d, the most reliable graph is a d-regular graph.*

Generally speaking, cubic graphs of high girth are good candidates for high-AC graphs [7]. Since RSRB can have a higher AC, they are also natural candidates for searching high-girth graphs. We pose this as a challenge.

**Challenge** **3.** *For a fixed average degree d and fixed number of vertices n, find graphs (not necessarily d-regular) with the highest possible girth.*

A *d*-regular graph with the smallest possible *n* for a given girth *g* is called a cage. The literature for looking for high-girth graphs is extensive; see [28,29] for an overview. There are powerful methods to perform computer searches for high-girth *d*-regular graphs [30,31]. As an example, consider $\left(2,6\right)$ RSRB graphs, whose average degree is d=3. Do $\left(2,6\right)$ semi-regular graphs give better cages than cubic graphs with the same number of vertices? The answer is, it depends. Note that that $\left(2,6\right)$ graphs can be obtained from 6-regular graphs by inserting a vertex in the middle of each edge; conversely, any $\left(2,6\right)$ graph yields a 6-regular graph by vertex contraction: just delete all 2-degree vertices. According to [28,32], a 6-regular 5-cage graph has 40 vertices. By vertex insertion, this yields a girth 10 $\left(2,6\right)$ semi-regular graph with 80 vertices. On the other hand, there are three cubic graphs having girth 10 and only 70 vertices [28]. So, the cubic is better for girth 10.

Similarly, a 6-regular cage of girth 6 has 62 vertices [28], which induces a (2,6) graph of girth 12 and 124 vertices. On the other hand, a cubic graph of girth 12 has at least 126 vertices [28,33]. So, a (2,6) semi-regular graph is superior for girth 12.

The techniques in this paper are rather general, and can be used to derive algebraic connectivity for many other random graph families. As an example, consider the “small-world”-type graph illustrated in Figure 1c. Start with a cycle of *n* vertices, then connect all odd-numbered vertices to each other at random. See Appendix A for the Matlab code that generates such a graph. The resulting graph has an average degree of 2.5. To compute its AC, as in the derivation of (5), we add a self-loop to even-numbered vertices so that each vertex has a degree of 3. We leave it as an exercise to the reader that the average walk-counting sequence $\phi_{s}$ has the generating function that satisfies

(18) $$\begin{array}{cc} A & =1+x^{2}\left(A\hat{A}+AB\right)\\ \hat{A} & =1+2x^{2}\hat{A}B\\ B & =1+xB+x^{2}AB\\ \phi_{o} & =1+x^{2}\phi_{o}\left(\hat{A}+2B\right)\\ \phi_{e} & =1+x\phi_{e}+2x^{2}\phi_{e}A\\ \phi & =\frac{1}{2}\phi_{o}+\frac{1}{2}\phi_{e}. \end{array}$$

Looking at the radius of convergence of the generating functions as before, we find that in the limit n→∞, its AC is asymptotic to the smallest root of the polynomial

$$\mu^{9}-24\mu^{8}+249\mu^{7}-1454\mu^{6}+5184\mu^{5}-11400\mu^{4}+14848\mu^{3}-10368\mu^{2}+3108\mu -136=0,$$

explicitly given by μ=0.0521926. This is validated using Monte Carlo simulations; the average AC of 1000 of such random graphs with n=1000 vertices is 0.0557 (std = 0.0029), i.e., a difference of about 7%. This is significantly higher than an RSR graph with $\left(p,d_{1},d_{2}\right)=\left(0.5,2,3\right)$, which also has an average degree of 2.5, but whose AC is μ∼0.044241.

## Data Availability

Data is contained within the article.

## Appendix A. Matlab and Maple Code

**RSRB model (Figure 1a):**

d1=2; d2=3; n1=30; n2=20;

bag1=mod([0:n1*d1-1], n1)+1;

bag2=mod([0:n2*d2-1], n2)+1+n1;

bag2=bag2(randperm(numel(bag2)));

G=graph(bag1, bag2);

plot(G);

**RSR model (Figure 1b):**

p=0.4; d1=2; d2=3; n=50;

n1=(1-p)*n; n2=p*n;

v1=mod([0:n1*d1-1], n1)+1;

v2=mod([0:n2*d2-1], n2)+1+n1;

bag=[v1, v2];

bag=bag(randperm(numel(bag)));

G=graph(bag(1:end/2),

bag(end/2+1:end));

plot(G);

**Small-world-like model (Figure 1c):**

n=52;

v1=[1:n];

v2=[2:n,1];

bag=[1:2:n];

bag=bag(randperm(numel(bag)));

v1=[v1,bag(1:end/2)];

v2=[v2,bag(end/2+1:end)];

G=graph(v1,v2);

t=(1:n)/n*2*pi;

plot(G, ’XData’, cos(t), ’YData’,

sin(t));

**Maple code to compute AC of RSR graphs:**
